# The relationship between perceived milk supply and exclusive breastfeeding during the first six months postpartum: a cross-sectional study

**DOI:** 10.1186/s13006-020-00310-y

**Published:** 2020-07-17

**Authors:** Ayyu Sandhi, Gabrielle T. Lee, Roselyn Chipojola, Mega Hasanul Huda, Shu-Yu Kuo

**Affiliations:** 1 Department of Pediatric and Maternity Nursing, Faculty of Medicine, Public Health and Nursing, Universitas Gadjah Mada , Yogyakarta, Indonesia; 2 School of Nursing, College of Nursing, Taipei Medical University, Taipei, Taiwan; 3 Applied Psychology, Faculty of Education, Western University, London, ON Canada

**Keywords:** Breastfeeding, Lactation, Human milk, Breast milk, Perception, Milk supply, Insufficient milk, Postnatal care, Infant feeding, Self-efficacy

## Abstract

**Background:**

Perceived milk supply is an important modifiable factor for optimal breastfeeding. However, little is known about maternal perception of milk supply or how it impacts breastfeeding practices. The aim of this study was to examine relationships of perceived milk supply, maternal breastfeeding self-efficacy, and skin-to-skin contact with early initiation and exclusive breastfeeding among mothers of infants less than 6 months of age in Indonesia.

**Methods:**

This was a cross-sectional study conducted in Yogyakarta City, Indonesia between August and October 2015. Maternal perception of milk supply was assessed using the Hill and Humenick Lactation Scale. Data on breastfeeding practices, and maternal and infant factors were collected using a structured questionnaire. Multiple regression and multivariate logistic regression analyses were performed to obtain estimates of associations.

**Results:**

Thirty four percent of mothers had initiated breastfeeding within an hour after birth, and 62.4% of mothers were exclusively breastfeeding. High levels of perceived breast milk supply were reported in mothers who practiced skin-to-skin contact or rooming-in with their infants, experienced positive infant sucking behavior, or had high breastfeeding self-efficacy (*p* < 0.05). Mothers with a higher level of perceived milk production (Odds Ratio [OR] 3.20; 95% Confidence Interval [CI] 1.76, 5.83) or practicing skin-to-skin contact (OR 2.36; 95% CI 1.13, 4.91) were more likely to exclusively breastfeed, while employed mothers were less likely to breastfeed their infants exclusively (OR 0.47; 95% CI 0.24, 0.93).

**Conclusions:**

In this study, skin-to-skin contact and breastfeeding self-efficacy are important determinants of perceived milk supply. Higher perception of milk supply was positively linked with exclusive breastfeeding. Our study highlights the importance of the assessment for mother’s perception of milk supply, maternal breastfeeding self-efficacy, and skin-to-skin contact in achieving optimal breastfeeding outcomes.

## Background

Breast milk is the optimal nutrition source for neonatal and infant wellbeing [1]. In 2012, the World Health Organization (WHO) established a global target to increase the rates of exclusive breastfeeding in the first 6 months to at least 50% by 2025 [2]. Recent estimates show that only 37% of infants aged 0 ~ 5 months are exclusively breastfed in low- and middle-income countries [3]. Specifically, the duration of exclusive breastfeeding remains a challenge in Indonesia with rates of 42% in 2012 and 37.3% in 2018 [4, 5]. Multilevel public health measures have been implemented in Indonesia to improve exclusive breastfeeding rates. For example, a legislation was enacted in 2009 that every baby is breastfed exclusively for the first 6 months of life, unless medically contraindicated [5]. Hospital breastfeeding support was also implemented by following the Ten Steps to Successful Breastfeeding of Baby-Friendly Hospital Initiative (BFHI), including facilitating skin-to-skin contact or rooming-in practice in Indonesian public hospitals [6]. In spite of legislation and hospital efforts, mothers’ perceptions of low milk supply and need for formula supplementation were the most common reasons for premature termination of breastfeeding in the immediate postpartum period and up to 6-months [7]. An in-depth understanding of mothers’ perception of milk supply and its relation to breastfeeding practices is necessary and will be helpful for developing effective healthcare strategies to improve exclusive breastfeeding rates in Indonesia.

Perception of insufficient milk supply is defined as a maternal belief that her breast milk production is inadequate for her infant’s needs [8]. Specifically, mothers often adopt some unreliable signs, such as infant satiety cues or infant crying, as the primary indicator of insufficient milk supply [9], instead of assessing the infant’s number of wet diapers and stools [10]. After delivery, mothers who perceive inadequate breast milk production tend to delay initiation of breastfeeding, wait until they believe their breast milk is ‘adequate’ [11], and may start to introduce unnecessary formula supplementation early [7]. Furthermore, studies suggest that approximately 25% ~ 73% of mothers engage in early unnecessary cessation of breastfeeding due to perceived low milk production [12, 13]. The early introduction of infant formula will result in a decrease in mothers’ milk production, which in turn interrupts their breastfeeding and further causes them to discontinue breastfeeding within the first 6 months postpartum.

Study findings have demonstrated that mothers’ perceptions of milk supply are modifiable, and early identification of mothers at risk is important for improving breastfeeding behavior [14]. Studies conducted in Japan and Taiwan found that mothers who were employed, had less than college education [15] or lacked breastfeeding confidence [12] had a significantly low perceived milk supply. The skin-to-skin contact between the mother and newborn is a significant factor in milk production and ejection, and may facilitate exclusive breastfeeding during the hospital stay [16]. However, an integrative review of 20 studies indicated that the prevalence of perceived low milk supply concerning socio-demographic variables or hospital breastfeeding behaviors has not been adequately addressed, and most studies were conducted in Western countries [14].

Maternal perception of milk insufficiency is a global challenge, and its impacts may vary across cultural contexts [14]. It is critical to examine maternal perception of milk supply and relevant factors in a developing country, like Indonesia, where exclusive breastfeeding is essential for infant health. To date, few attempts have been made to identify associated factors in maternal perception of milk supply in Indonesian women, particularly in the community setting where mothers often experience difficulties in breastfeeding after hospital discharge. In response to the WHO’s call to increase exclusive breastfeeding and the current low rates of exclusive breastfeeding in Indonesia, the objectives of this study were to identify factors associated with mothers’ perceived milk supply and investigate associations between this perception and breastfeeding practices such as early initiation of breastfeeding and exclusive breastfeeding in Indonesia.

## Methods

### Design

A cross-sectional study was conducted from August to October 2015 in Yogyakarta City, Indonesia. In total, five public health centers (PHCs) with low exclusive breastfeeding rates, ranging from 14.8 to 25.0%, were selected based on the available estimates in 2009 [17]. Convenience sampling was used to recruit eligible participants who were approached at their residence based on name lists provided by the PHCs. The study was approved by the institutional review board of Universitas Gadjah Mada, Indonesia. Written informed consent was obtained from each mother after the study purpose was explained.

### Participants

Study participants were mother-infant dyads with infants under 6 months of age. Mothers were included in the study if they met the following criteria: were aged ≥17 years, had a singleton pregnancy, had given birth at ≥37 weeks of gestation, had an infant with a birth weight of ≥2500 g, and understood and spoke the Indonesian language. Mothers were excluded if they had a history of breast surgery, postpartum complications, or their infants had any neonatal anomaly or any admission history to a neonatal intensive care unit. The sample size was estimated based on a prior study on postpartum mothers [18] with an effect size of 0.30 for perception of milk supply. With a significance level of 0.05 and 80% power in a two-sided *t*-test, the sample size of 176 was needed. Considering an estimated 30% incompletion rate, a total of 230 mothers were recruited for this study.

### Measurements

Participants completed the self-reported questionnaires after signing the informed consent. It took 20 to 30 min for each participant to complete the questionnaire. To avoid any missed items, all questionnaires were checked upon their completion.

#### Translation process

The Hill and Humenick (H&H) Lactation Scale [8], Breastfeeding Self-Efficacy Scale Short Form [19], and infants’ sucking behaviors [20] were translated into Bahasa Indonesia following guidelines of Wild et al. [21]. First, a bilingual translator with a qualified health professional background translated the original questionnaires from English into Bahasa Indonesia (forward translation). Two Bahasa Indonesia-speaking experts in the field of postpartum care and breastfeeding then reviewed the translated version for content and precision of the wording to Indonesian culture (reconciliation). Second, the Indonesian version was translated back into English by another independent bilingual qualified translator (back translation). The original and back-translated questionnaires were compared for clarity and consistency by the researchers. Third, the Indonesian versions of the questionnaires were administered to 10 Indonesian mothers to ensure the applicability of the translated questionnaires to the Indonesian population.

#### Perceived milk supply

The maternal perception of milk supply was assessed using the H&H Lactation Scale [8], a 20-item questionnaire with a 7-point Likert scale from 1 (strongly disagree) to 7 (strongly agree). Reverse scoring was applied to items 3 (“Even if I can breastfeed, I would rather not be breastfeeding”), 8 (“I am so upset about breastfeeding problems that I become upset at the thought of breastfeeding”), 12 (“My baby would be hungry if I did not use formula along with breastfeeding”), 13 (“I believe that following breastfeeding with a bottle is how to find out if baby get enough”), 14 (“I would describe my baby as being fussy after breastfeeding”), and 15 (“I feel have to give formula after breastfeeding to satisfy my baby”). Total scores ranged 20 ~ 140, with higher scores representing a higher perceived milk supply. The total score was re-grouped into dichotomous categories using the mean score of the H&H Lactation Scale for subsequent analyses. Perceived milk supply was originally assessed as a continuous variable and was dichotomized into low and high perceived milk supply using the mean score for subsequent analyses in order to identify the high-risk group as recommended in previous literature [14]. No missing value was found in this scale in this study. Cronbach’s alpha values of the original study ranged 0.91 ~ 0.92 [8]. In this study, the content validity index (CVI) of the Indonesian version of the H&H Lactation Scale was 0.99, and Cronbach’s alpha was 0.80.

#### Breastfeeding self-efficacy

The Breastfeeding Self-Efficacy Scale (BSES) Short Form (SF) is a 14-item self-reported questionnaire refined from the original BSES [19], that measures breastfeeding self-efficacy on a 5-point Likert scale from 1 (not at all confident) to 5 (very confident). Total possible scores range 14 ~ 70, with higher scores indicating higher levels of breastfeeding self-efficacy. Cronbach’s alpha from the original study was 0.94 [19]. In this study, the CVI of the Indonesian version of the BSES-SF was 0.99, and Cronbach’s alpha was 0.90. Breastfeeding self-efficacy, converted to a dichotomous variable with the cut-off point of 50, has shown to be a valuable predictor for identifying mothers who need breastfeeding support in previous study [22]. The breastfeeding self-efficacy was divided into high (> 50) and low (≤50) in the current study.

#### Infant sucking behavior

Mothers were asked to assess infants’ sucking behavior as defined by Mizuno et.al [20]. Five types of infants’ sucking behaviors were classified as follows: type 1: infant vigorously and promptly grasps the nipple and sucks energetically; type 2: infant is so excited and active at the breast, that he/she alternatively grasps or loses the breast; type 3: infant sucks the breast slowly and reluctantly; type 4: infant usually spends a few minutes of mouthing, tastes small amounts of milk before settling down, and breastfeeds very well; and type 5: infant sucks the breast for a few minutes and then rests a few minutes and repeats the behavior pattern alternatively thereafter. Infants’ sucking behaviors were regrouped into two categories of positive (types 1, 4, or 5), and negative (type 2 or 3) for subsequent analyses. The CVI of the Indonesian version of infant sucking behavior was 0.95.

#### Breastfeeding practices

In this study, we adopted the WHO definitions of breastfeeding practices [23]. The definitions are as follows.

##### Early breastfeeding initiation

Early breastfeeding is defined as the mother putting her baby to the breast within 1 h of birth. In this study, initiation of breastfeeding was self-reported by mothers based on their recall.

##### Exclusive breastfeeding

Exclusive breastfeeding is defined as the mother feeding her baby exclusively with breast milk, directly from the breast or expressed, and with no additional foods in the previous 24 h. Mothers were asked to recall what food was given to their infants until the day prior to the survey.

##### Predominant breastfeeding

Predominant breastfeeding is defined as the infant receiving breast milk as the predominant source of nourishment with certain liquids (e.g., water, water-based drinks, fruit juice, and oral rehydration solution, drops, or syrup).

##### Demographic variables and health indicators

Information on demographic characteristics (age, educational level, employment status, and religion), obstetric factors (parity, delivery type, and delivery location), hospital factors (rooming-in practice and skin-to-skin contact), and infant factors (age, gender and birth weight) were collected using structured questionnaires.

### Data analysis

Data were analyzed using the Statistical Package for the Social Sciences for Windows (version 17.0; SPSS, Chicago, IL). Descriptive statistics including the percentage, frequency, mean (M), and standard deviation (SD) were used to report the distribution of the variables. A *t*-test or ANOVA were used to compare the scores between maternal perception of milk supply and potential factors, including maternal characteristics (age, educational level, employment status, religion), obstetric factors (parity, delivery type, delivery location), hospital procedures (rooming-in practice, skin-to-skin contact), infant factors (infant’s age, gender, birth weight, infant sucking behavior), and breastfeeding self-efficacy. We used multiple regression to examine the determinants of perceived milk supply. Potential predictors for maternal perception of milk supply were included in the regression model if their bivariate association with perceived milk supply had a *p*-value of ≤0.10. Multicollinearity between independent variables was examined using the Variance Inflation Factor and Tolerance index. A multivariate logistic regression analysis was performed to estimate relationships between perceived milk supply and breastfeeding practices. We first ran the univariate logistic analyses and then included only those variables with *p* ≤ 0.10 or with clinical meaningfulness in the multivariable logistic model.

## Results

In total, 250 eligible mothers with infants aged 0 ~ 5 months were invited to participate, and 237 completed the questionnaires. The response rate was 94.8%.

### Characteristics of study participants and breastfeeding practices

The average age of the mothers was 30 (SD = 5.5) years. The majority of the participants had at least high school education (67.1%), were not working (73.0%), and were Muslims (90.3%) (Table 1). More than half of the mothers were multiparous (59.9%), had a normal vaginal delivery (73.0%), and had delivered in the hospital (68.8%). Most of the mothers practiced rooming-in (63.7%) and skin-to-skin contact (70.0%) within the first hour after delivery. The average age of the infants was 2.7 (SD = 1.6) months, and they had an average weight of 3172 g (SD = 397). Slightly more than half of the babies were girls (53.6%), and most showed positive sucking behaviors (84.4%) (Table 1).

**Table 1:** Characteristics of study participants (*N* = 237)

Variable	***n***	***%***
**Age (years), Mean (SD)**	30.0 (5.5)	
≤30	129	54.4
> 30	108	45.6
**Educational level**		
High school and less	159	67.1
College and above	78	32.9
**Employment status**		
Not working	173	73.0
Working	64	27.0
**Religion**		
Islam	214	90.3
Other	23	9.7
**Parity**		
Primiparous	95	40.1
Multiparous	142	59.9
**Mode of Delivery**		
Vaginal delivery/ Instrumental vaginal delivery	173	73.0
Cesarean delivery	64	27.0
**Delivery location**		
Hospital	163	68.8
Public health center/midwifery clinic	74	31.2
**Rooming-in**		
No	86	36.3
Yes	151	63.7
**Skin-to-skin contact**		
No	71	30.0
Yes	166	70.0
**Infant’s age (months), Mean (SD)**	2.7 (1.6)	
0 ~ 1	56	23.6
2 ~ 3	95	40.1
4 ~ 5	86	36.3
**Infant’s gender**		
Girl	127	53.6
Boy	110	46.4
**Infant’s birth weight (g)**		
≤ 3000	95	40.1
> 3000	142	59.9
**Infant sucking behavior**		
Negative	37	15.6
Positive	200	84.4

Note: *SD* Standard Deviation

Approximately one-third of the mothers (34.2%) initiated breastfeeding in the first hour, 62.4% breastfed their babies exclusively at 0–5 months, and 37.6% practiced predominant breastfeeding (Table 2). The mean score of perceived milk supply was 122.4 (SD = 10.0), with a range of 81 ~ 140. The mean score of perceived milk supply among mothers of infants aged 4 ~ 5 months was 123.6 (SD = 10.0) with the range of 98 ~ 140, and this was the highest mean score across infant age groups. The participants’ mean score on breastfeeding efficacy was 56.4 (SD = 7.2) with a range of 26 ~ 70. High breastfeeding self-efficacy (86.9%) was reported among most participants (Table 2).

**Table 2:** Characteristics of perceived milk supply, breastfeeding self-efficacy, early initiation of breastfeeding, and exclusive breastfeeding (*N* = 237)

Variable	***n (%)***	***Mean (SD)***	***Min***	***Max***
**Early initiation of breastfeeding**	81 (34.2)			
**Predominant breastfeeding**	89 (37.6)			
**Exclusive breastfeeding (0 ~ 5 months)**	148 (62.4)			
0 ~ 1 month	39 (69.6)			
2 ~ 3 months	59 (62.1)			
4 ~ 5 months	50 (58.1)			
**Perception of the milk supply, Mean (SD)**		122.4 (10.0)	81	140
0 ~ 1 month, Mean (SD)		122.0 (10.8)	81	139
2 ~ 3 months, Mean (SD)		121.4 (9.4)	96	139
4 ~ 5 months, Mean (SD)		123.6 (10.0)	98	140
**Breastfeeding self-efficacy, Mean (SD)**		56.4 (7.2)	26	70
≤ 50	31 (13.1)		26	50
> 50	206 (86.9)		51	70

Note: *SD* Standard Deviation, *Min* Minimum, *Max* Maximum

### Associations between maternal/infant factors with perceived milk supply

Results of the univariate analysis indicated that rooming-in, skin-to-skin contact, breastfeeding self-efficacy, and infant sucking behavior were significantly associated with perceived milk supply (*p* < 0.05), while mothers’ age, educational level, employment, parity, mode of delivery, delivery location, and infants’ age, gender, and birth weight were not (Table 3). Mothers’ perceptions of milk supply were not significantly different for each infant age group (*p* > 0.05). Considering that *p* values of employment and parity were < 0.10, both variables were included in the multivariate analysis. In the multiple linear regression model, skin-to-skin contact and breastfeeding self-efficacy remained significantly associated with maternal perception of milk supply (*p* < 0.02) (Table 4). *R*^2^ and adjusted *R*^2^ values for this model were 0.21 and 0.19, respectively. The variance inflation factors for the variables (1.04 ~ 1.08) were not above the value of 10, and the tolerance values for all variables in model (0.93–0.96) were not < 0.16, indicating a lack of multicollinearity between variables.

**Table 3:** Distribution of perceived milk supply (*N* = 237)

Variable	***n***	***%***	Perception of the milk supply				
			***Mean***	***SD***	***Min***	***Max***	***p value***
**Age (years)**							
≤ 30	129	54.4	122.3	9.7	96	139	0.90 ^a^
> 30	108	45.6	122.4	10.3	81	140	
**Educational level**							
High school and less	159	67.1	122.1	9.5	95	140	0.57 ^a^
College and above	78	32.9	122.9	10.9	81	138	
**Employment status**							
Not working	173	73.0	123.1	9.7	81	139	0.07 ^a^
Working	64	27.0	120.5	10.5	98	140	
**Religion**							
Islam	214	90.3	122.3	9.7	95	140	0.77 ^a^
Other	23	9.7	122.9	12.1	81	135	
**Parity**							
Primiparous	95	40.1	120.9	10.4	96	139	0.06 ^a^
Multiparous	142	59.9	123.4	9.6	81	140	
**Mode of Delivery**							
Vaginal delivery/IVD	173	73.0	122.9	9.6	95	140	0.17 ^a^
Cesarean delivery	64	27.0	120.9	10.8	81	139	
**Delivery location**							
Hospital	163	68.8	121.9	10.6	81	140	0.27 ^a^
Public health center/midwifery clinic	74	31.2	123.3	8.4	100	138	
**Rooming-in**							
No	86	36.3	120.4	9.4	98	140	0.02 ^a^
Yes	151	63.7	123.5	10.1	81	139	
**Skin-to-skin contact**							
No	71	30.0	119.2	10.7	81	139	0.001 ^a^
Yes	166	70.0	123.7	9.3	95	140	
**Breastfeeding self-efficacy**							
≤ 50	31	13.1	111.8	10.7	81	132	0.0001 ^a^
> 50	206	86.9	123.9	8.9	95	140	
**Infant’s age (months)**							
0 ~ 1	56	23.6	122.0	10.8	81	139	0.31 ^b^
2 ~ 3	95	40.1	121.4	9.4	96	139	
4 ~ 5	86	36.3	123.6	10.0	98	140	
**Infant’s gender**							
Girl	127	53.6	121.9	10.2	81	139	0.50 ^a^
Boy	110	46.4	122.8	9.8	95	140	
**Infant’s birth weight (g)**							
≤ 3000	95	40.1	121.8	9.8	96	140	0.51 ^a^
> 3000	142	59.9	122.7	10.1	81	139	
**Infant sucking behavior**							
Negative	37	15.6	119.4	9.4	98	134	0.05 ^a^
Positive	200	84.4	122.9	10.0	81	140	

Note: *IVD* instrumental vaginal delivery, *SD* Standard Deviation, *Min* Minimum, *Max* Maximum; ^a^t-test; ^b^one-way ANOVA

**Table 4:** Multiple linear regression analysis of perceived milk supply (*N* = 237)

Variable	***n***	***%***	***β***	***SE***	***Standardized β***	***p value***
**Intercept**			89.9	5.37		< 0.0001
**Employment status**						
Not working	173	73.0	Reference			
Working	64	27.0	−0.96	1.36	−0.04	0.48
**Parity**						
Primiparous	95	40.1	Reference			
Multiparous	142	59.9	0.89	1.21	0.04	0.46
**Rooming-in**						
No	86	36.3	Reference			
Yes	151	63.7	1.69	1.25	0.08	0.18
**Skin-to-skin contact**						
No	71	30.0	Reference			
Yes	166	70.0	3.20	1.31	0.15	0.02
**Breastfeeding self-efficacy**						
≤ 50	31	13.1	Reference			
> 50	206	86.9	10.78	1.81	0.37	< 0.0001
**Infant sucking behavior**						
Negative	37	15.6	Reference			
Positive	200	84.4	2.10	1.63	0.08	0.20

Note: *SE* standard error

### Association between perceived milk supply and early initiation of breastfeeding

Logistic regression analyses were used to examine how perceptions of the maternal milk supply were associated with early initiation of breastfeeding. We used the mean score to categorize perceived milk supply score into two groups (scores of ≤122 and > 122). In the univariate analysis, the odds ratio (OR) of perceived milk supply was 1.46 (95% Confidence Interval [CI] 0.85, 2.50, *p* > 0.05). Results of the multivariate analyses indicated that caesarean delivery (OR 0.13; 95% CI 0.04, 0.41, *p* = 0.0001) and skin-to-skin contact (OR 2.81; 95% CI 1.14, 6.91, *p* = 0.024) were significantly associated with early initiation of breastfeeding. Neither perceived milk supply, education level, employment status, delivery location, nor rooming-in practice were associated with early initiation of breastfeeding (Table 5).

**Table 5:** Logistic regression analysis of perceived milk supply, early initiation of breastfeeding and exclusive breastfeeding (*N* = 237)

Variable	Early initiation of breastfeeding			Exclusive breastfeeding		
	n	%	OR (95% CI)	n	%	OR (95% CI)
**Univariate analysis**						
**Perceived milk supply**						
≤ 122	35	29.9	1.00	56	47.9	1.00
> 122	46	38.3	1.46 (0.85, 2.50)	92	76.7	3.58 (2.05, 6.25)***
**Multivariate analysis**						
**Perceived milk supply**						
≤ 122	35	29.9	1.00	56	47.9	1.00
> 122	46	38.3	1.84 (0.93, 3.6)	92	76.7	3.20 (1.76, 5.83)***
**Educational level**						
High school and less	50	31.4	1.00	105	66.0	1.00
College and above	31	39.7	1.84 (0.96, 3.64)	43	55.1	0.73 (0.38, 1.39)
**Employment status**						
Not working	55	31.8	1.00	117	67.6	1.00
Working	26	40.6	1.53 (0.75, 3.15)	31	48.4	0.47 (0.24, 0.93)*
**Mode of delivery**						
Vaginal delivery	77	44.5	1.00	117	67.6	1.00
Cesarean delivery	4	6.3	0.13 (0.04, 0.41)***	31	48.4	0.82 (0.38, 1.79)
**Delivery location**						
Hospital	47	28.8	1.00	96	58.9	1.00
Public health center/midwifery clinic	34	45.9	1.36 (0.71, 2.60)	52	70.3	1.14 (0.57, 2.28)
**Rooming-in**						
No	22	25.6	1.00	47	54.7	1.00
Yes	59	39.1	1.21 (0.61, 2.38)	101	66.9	1.12 (0.60, 2.08)
**Skin-to-skin contact**						
No	8	11.3	1.00	31	43.7	1.00
Yes	73	44.0	2.81 (1.14, 6.91)*	117	70.5	2.36 (1.13, 4.91)*

Note: *OR* odds ratio, *CI* confidence interval; * *p* < 0.05; ** *p* < 0.01; *** *p* < 0.0001

### Association between perceived milk supply and exclusive breastfeeding

In the univariate analysis, perceived milk supply was significantly associated with exclusive breastfeeding (OR 3.58; 95% CI 2.05, 6.25, *p* = 0.0001). In the multivariate model, high levels of perceived milk supply (OR 3.20; 95% CI 1.76, 5.83, *p* = 0.0001) or skin-to-skin contact (OR 2.36; 95% CI 1.13, 4.91, *p* = 0.022) were significantly associated with exclusive breastfeeding, while working mothers were less likely to practice exclusive breastfeeding during 0–5 months postpartum (OR 0.47; 95% CI 0.24, 0.93, *p* = 0.029). Variables of the educational level, mode of delivery, delivery location, and rooming-in were no longer significant (Table 5).

## Discussion

The present study is the first study to investigate mothers’ perceptions of milk sufficiency and how their perception was associated with early initiation of breastfeeding and exclusive breastfeeding among mother-infant dyads in Indonesia. The study revealed that mothers who practiced skin-to-skin contact or had high self-efficacy in breastfeeding were more likely to have higher levels of perceived milk supply. Higher levels of perceived milk supply were linked to higher exclusive breastfeeding rates but not to early initiation of breastfeeding. Our study highlights the importance of maternal perception of the milk supply on optimal breastfeeding outcomes.

The rates of exclusive breastfeeding (62.4%) in our study, to our surprise, were higher than we expected from the statistics provided by these study PHCs (14.8 to 25.0%) and were higher than the national estimates (37.3%) in Indonesia [4]. Nevertheless, the result of this study was consistent with the rates of exclusive breastfeeding (63.3%, 60%) reported in two recent studies conducted in Yogyakarta regions [24, 25], in which the definition for exclusive breastfeeding by WHO during the first 6 months postpartum was adopted. The relatively high exclusive breastfeeding rates in Yogyakarta regions might be related to multilevel breastfeeding interventions launched since 2012, including peer breastfeeding support and lactation counseling [26]. Therefore, these interventions should continue in Yogyakarta regions, and replications of the interventions for other regions with relatively low rates of exclusive are necessary.

### Mothers’ perception of milk supply

We found that perception of milk supply was an important factor associated with exclusive breastfeeding in Indonesian mothers. This finding is consistent with previous studies in Turkey [27] and Mexico [9], which reported that the perception of insufficient milk was prevalent in postpartum mothers who did not practice exclusive breastfeeding. In Indonesian culture, mothers tend to believe that small breasts produce smaller amounts of breast milk and older mothers’ breast milk is of lower quality [28], which in turn, impact mothers’ perceptions of milk insufficiency. The mothers in our study had a higher perception of breast milk (mean score 122.4 in the Hill and Humenick Lactation Scale) in the first 6 months postpartum compared to perceptions of breast milk supply in a previous study conducted in Taiwan when women were 1 week postpartum (mean scores 106.8–108.2) [15]. It is possible that levels of perceived milk supply vary at different postpartum stages, as breast milk might not be fully established at 1 week postpartum [10]. Despite our study having indicated that mothers’ perception of milk supply was not significantly different for each infant age group, assessing potential changes in maternal perception of the milk supply over different postpartum stages warrants further research.

### Factors associated with perceived milk supply

In this study, Indonesian mothers who practiced skin-to-skin contact not only reported significantly high levels of breast milk sufficiency but also initiated breastfeeding early and breastfed infants exclusively in the first 6 months postpartum. Specifically, skin-to-skin contact produces massage-like movements (e.g., touching of the areola and breast skin) to stimulate lactation [29] with increased opportunities for infant suckling, resulting in a surge of plasma oxytocin which triggers the let-down reflex [30]. Experiencing the let-down reflex accompanied by baby suckling also makes a mother perceive that her milk supply is sufficient for her infant [14]. Studies of Egyptian and US mothers also found the positive effect of skin-to-skin contact on early initiation of breastfeeding [31] and exclusive breastfeeding [32]. Early skin-to-skin contact is important for optimal breastfeeding practices and therefore, it is essential to support mothers practicing skin to-skin contact at an early stage.

Consistent with the findings of a previous study [33], we found that mothers who practiced rooming-in tend to have higher perception of sufficient milk supply. It is possible that placing the infant close to the mother enables the mother to respond in a timely manner when her infant shows signs of hunger. Uninterrupted mother-infant interactions and close contact also encourage breastfeeding on demand, which in turn, results in more frequent infant suckling that promotes breast milk production [34]. Our findings also confirmed the positive effect of breastfeeding self-efficacy on perceived milk sufficiency as reported by earlier research in Japanese mothers [12]. A recent meta-analysis indicates that educational interventions targeting breastfeeding self-efficacy may alleviate perceptions of breast milk insufficiency and improve breastfeeding outcomes [35]. These findings once again support that mothers’ participation in Baby-Friendly practice (i.e., skin-to-skin contact and rooming-in) had better perception of breast milk sufficiency [36].

### The relation between perceived milk supply and breastfeeding practice

In the present study, we found that mothers who had a high perception of sufficient breast milk were more likely to exclusively breastfeed, but tended not to practice early initiation of breastfeeding. Prior studies conducted in India and Bangladesh [37–39] reported that mothers with a perception of insufficient milk tended to delay initiation of breastfeeding. The different findings might be partly explained by the existence of cultural beliefs about breast milk. For example, the belief that colostrum is harmful for the newborn [38, 39] or the early introduction of prelacteal feeding might infect the baby’s gut may lead mothers to delay initiation of breastfeeding [39]. Such cultural beliefs about breast milk are common for delayed breastfeeding in Indonesia [40].

The majority (90.3%) of our participants were Islamic, who obeyed the teaching of breastfeeding babies for first 2 years (The Quran verse 2:233). The infant’s father has the responsibility to support breastfeeding under all circumstances. Providing a shelter and financial support for breastfeeding is required should a divorce occurs (verses 2:233 and 65:6) [41]. It is possible that such a religious belief contributes to the high rate of exclusive breastfeeding in our study.

### Relevance to clinical practice

Results of this study have important implications for healthcare practices both in clinical and community settings. Health professionals, especially nurses, might consider assessing perceived milk supply in antenatal or postpartum care to identify and provide support promptly to mothers who perceive they have insufficient milk. Antenatal breastfeeding interventions based on breastfeeding self-efficacy have been suggested as an effective strategy for increasing mother’s confidence [42, 43] and could be used to eliminate barriers due to misconceptions. Professional support in hospitals (e.g., skin-to-skin contact, the rooming-in practice, and lactation counselling) guided by the Ten Steps to Successful Breastfeeding will be helpful for mothers during hospital stay when mothers’ decisions on weaning or supplementing breastfeeding are made [44, 45]. It is also important to collaborate with multiple sectors in health systems, service providers, community health workers, stakeholders, and policymakers [35] to support women with breastfeeding needs in order to ensure successful and continuous breastfeeding.

### Limitations

Several limitations should be considered when interpreting results of this study. First, the cross-sectional study design only allows an interpretation of the correlations between perceived milk supply and breastfeeding practices and thus causal effects among the variables cannot be drawn. Researchers could consider conducting longitudinal studies to examine potential causal relationships in future studies. Second, our sample only contained healthy women and was conducted in the Java island region of Yogyakarta city in Indonesia focusing on mothers receiving care from the PHCs, not the in-hospital mothers. Future studies need to include mothers at risk and mothers from large number of PHCs or hospitals in Indonesia to increase the heterogeneity of the sample. Third, the self-reported data pertaining to the postpartum period (e.g., early initiation of breastfeeding, skin-to-skin contact) were collected retrospectively and thus may be subject to recall bias. In this study, mothers’ perception of their milk supply was measured using a self-reporting scale. The actual amount of milk supply was not measured due to the lack of available tools in this study. Future researchers could consider exploring the associations between mothers’ perception of their milk supply and their actual amount of milk supply and examine whether the related factors remain associated with maternal perception of milk supply and actual milk supply.

## Conclusions

Mothers who practiced skin-to-skin contact and had high self-efficacy in breastfeeding tended to have higher levels of perceived milk supply, and a perception of sufficient milk supply was linked to higher odds of exclusive breastfeeding. Our study highlights the importance of the prompt assessment for mother’s perception of milk supply during the postpartum stage. These findings are helpful in guiding the development of effective interventions in clinical and community settings. The health care professionals can then provide these interventions to assist optimal breastfeeding practices for mothers in developing countries where exclusive breastfeeding is essential for infant health.

## Data Availability

The datasets used and analyzed during the current study are available from the corresponding author on reasonable request.
